# Comparative evaluation of the healing potential of excision wound using Nano- formulations of silver, gold, or their mixture: *In-silico* and *in vivo* approaches

**DOI:** 10.1371/journal.pone.0347682

**Published:** 2026-04-30

**Authors:** Abeer A. Mabrouk, Sameh H. Ismail, Ahmed G. Soliman, Mervat M. Omran

**Affiliations:** 1 Department of Biochemistry, Egyptian Drug Authority (NODCAR), Cairo, Egypt; 2 Egypt Nanotechnology Centre, Cairo University, El-Sheikh Zayed, Giza, Egypt; 3 Agricultural Biochemistry Department, Faculty of Agriculture, Ain Shams University, Shoubra El-Kheima, Cairo, Egypt; 4 Cancer Biology Department, National Cancer Institute - Cairo University, Cairo, Egypt; 5 Department of Obstetrics and Gynecology, University of Chicago, Chicago, Illinois, United States of America; Brandeis University, UNITED STATES OF AMERICA

## Abstract

The medical use of metal nanoparticles is recommended to reduce adverse effects and improve curative action when compared to their pure ions. Current research discussed the healing effect of topical formulations of silver, gold, and their mixtures of nanoparticles on excision wounds in rat models. The physicochemical factors of the prepared nanoparticles were measured by **dynamic light scattering** (DLS), Zeta potential, **atomic force microscopy** (AFM), and transmission electron microscopy (TEM). Thirty-two albino rats were divided into six groups: wound untreated, silver nanoparticles (AgNps) treated, gold nanoparticles (AuNps), silver-gold nanoparticles (Ag/AuNps 1:1) mixture treated, and fusidic acid (reference drug) treated groups, respectively. Management was performed daily for 21 days. In all groups, the percentages of contraction area and histological investigation of the wound area were investigated. Myeloperoxidase (MPO), nitric oxide (NO), vascular endothelial growth factor (VEGF), hydroxyproline, hexosamine, and collagen deposition using **Masson’s trichrome** stain (MT) as a biomarker of wound healing activity were measured. An in-silico study investigated the molecular interaction of the metal nanoformulations with various protease enzymes involved in inflammation and the **absorption, distribution, metabolism, excretion, and toxicity** (ADMET) properties of the examined metals. The AgNPs and AuNPs have efficient wound-repairing activity, as evident from the wound shrinkage and enhanced wound healing. Metal nanoformulations have effective wound healing activity by enhancing angiogenesis and collagen formation.

## 1 Introduction

The wound healing process comprises four ordered biological sections: homeostasis, inflammation, proliferation, and remodeling. Abnormalities in any of these stages may cause a long-term wound repairing process resulting in poorly repaired skin tissue [1,2]. Firstly, once a fibrin clot is made, it represents a scaffolding for the immigration of inflammatory cells to the wound. Infiltrate cells that reach the wound site: Polymorphonuclears (PMNs) start and are followed by macrophages. These represent the main source of cytokines, principally TNF-α is involved in inflammation [3]. They are responsible for the degeneration of the wound matrix and induction of neutrophil migration; these released factors from neutrophils cause a delay in the wound repair process. Macrophages contribute to wound debridement and perform a vital role in the regulation of angiogenesis, matrix deposition, and remodeling. During the proliferative phase, fibroblasts seem to be on the scene to create the required collagen and endothelial cells for the angiogenesis stage [4,5]. The creation of collagen is based on regulatory factors. For instance, adequate oxygenation, supplies of cofactors, nutrients, and local wound surroundings. Finally, remodeling begins during the fibroplasia stage. The wound strength is evaluated by the quality and extent of precipitated collagen. Epithelialization starts after injury within one day and stays for many days based on the wound size [3]. However, any defects or abnormalities in the wound healing process may cause dangerous microbial infections and a weakening in wound healing Arneth et al., [6]. In addition, the count of antibiotic-resistant microorganisms is widely increasing, forcing the scientific community to continually search for novel drugs. It has been stressed over the years to search for an alternative option in wound therapy, such as nanoparticle preparations [7,8]. Nanoparticles own physicochemical properties superior to bulk resources because of their huge surface to volume ratio, greater stability, bioactivity, bioavailability, controlled PS, controlled release of loaded remedies, and precise targeting. Moreover, nanoparticles exert an unbelievable potential for drug delivery because of their ability to cross cells, tissues, and organs, enhancing the poor bioavailability and great toxicity of current medicines [9]. Concerning metallic and metal oxide NPs, most studies have been conducted on silver, gold, and zinc compounds because of their unique characteristics such as antibacterial activity and increased skin penetration [10,11]. Silver nanoparticles (AgNPs) can avoid the restrictions of standard silver medications (for example, sulfadiazine). Because of their augmented surface-to-volume ratio, AgNPs are more effective at decreased concentrations, thus reducing their toxicity. Silver nanoparticles can modify anti-inflammatory cytokine discharge to accelerate wound closure and reduce scarring. Through stimulating the production of myofibroblasts from normal fibroblasts, AgNPs induce wound contraction, thus accelerating the healing process [12]. Furthermore, AgNPs promote epidermal re-epithelialization by the proliferation and repositioning of keratinocytes. Typically, gold nanoparticles (AuNPs) are considered an alternative option in wound therapy because of their chemical stability and ability to absorb near-infrared light, in addition to being reasonably easily synthesized [13]. Furthermore, AuNP gels improve thermoresponsiveness, as described by Arafa et al. [14], Who investigated their antibacterial and healing characteristics *in vitro* and *in vivo*. Furthermore, AuNPs inhibit the generation of reactive oxygen species, acting as antioxidants, helping the healing process [15]. Marza et al. [16] showed the effect of AuNPs in Vaseline mixtures at 6, 12, and 18 wt% for 14 days on wound healing, The outcomes revealed that the formulations induced angiogenesis and fibroblast proliferation, accelerating wound closure, without causing cell toxicity [16]. Furthermore, the study of Shanmugasundaram et al. [8] demonstrated antibacterial activity of the prepared Ag, Au, and Ag/Au alloy nanoparticles against a wide range of human bacterial pathogens, such as gram-positive and gram-negative bacteria. The zone of inhibition for antibiotic activity against gram-positive bacteria ranged from 8.33 mm in diameter, and for gram-negative bacteria from 7.66 to 15.16 mm. The antibacterial activity results clearly showed that Ag, Au, and Ag/Au nanoparticles dominate more inhibition of gram-negative bacterial growth than gram-positive bacteria.

This study aimed to investigate the healing effect of the formulated AgNPs, AuNPs, or their mixture (AgNPs/AuNPs; 1:1) containing a gel on an excision wound in female albino rats. Furthermore, histological investigations of skin have been conducted to assess histological variations. A molecular docking method was implemented to investigate the molecular interaction of the prepared metal nano-formulations with various protease enzymes (collagenase, elastase, and trypsinase) and their ADMET properties.

## 2 Materials and methods

### 2.1 Materials

#### 2.1.1 Chemicals used for synthesis.

Trisodium citrate (TSC), Carbopol 940, silver nitrate (AgNO_3_), and gold chloride (Aucl_3_) were purchased from Sigma Chemical Co. (St Louis, MO, USA). Fucidin cream 2% is fusidic acid obtained by Minapharm Company, Egypt.

#### 2.1.2 Experimental animals.

The present study was carried out on forty female albino rats weighing 160–180 g. The standard NODCAR strategies were applied during animal handling. Animals had free access to food and water *ad libitum*. They were kept at 21–24˚C and relative humidity with a 12-h light–dark cycle. Animals were handled quietly; squeezing, stress, and rough maneuvering were avoided. The NODCAR Animal Ethnics Committee approved the protocol with approval number NODCAR/ΙΙ/22/2022.

### 2.2 Methods

#### 2.2.1 Synthesis of silver nano-metal/Carbopol hybrid nano-gel.

Silver nano-metal/Carbopol hybrid nano-gel is synthesized in two steps. The first step is the synthesis of silver nanoparticles, and the second step is the hybridization of carbopol gel with silver nanoparticles to prepare silver nanometal/Carbopol hybrid nano-gel. In a typical synthesis of silver nanoparticles, TSC is used as a precipitating agent according to the method of Ismail et al. [17]. The first step is the preparation of silver nanoparticles by the precipitation method. Typically, the 0.001M of silver nitrate was dissolved in 100 mL of doubly deionized water; then 0.1 M TSC was added dropwise. The mixture solution was subjected to ultra-sonication using Hielscher UP400S (400 W, 24 kHz) at an amplitude of 90% and a cycle of 0.9 for 6 minutes at a temperature of 120°C, until a pale-yellow colloidal solution of silver nanoparticles was obtained. Then, the resulting solution was cooled to room temperature, avoiding light incidence. The second step is the synthesis of silver nano-metal/Carbopol hybrid nano-gel by the sono-chemical method. Typically, 2g of Carbopol 940 was dissolved in 500 ml of deionized water, and added to the prepared silver nanoparticles and sonicated with a propeller device, amplitude 92, and cycle 92% for 15 minutes; then 200 ml of triethylamine was added with continuous sonication until pale yellow gel formation.

#### 2.2.2 Synthesis of gold nano-metal/Carbopol hybrid nano-gel.

The gold nano-metal/Carbopol hybrid nano-gel was synthesized in two steps according to the method of Turkevich et al. [18] with some modifications. Initially, 0.0024mM of gold chloride was dissolved in 60 ml of doubly distilled deionized water, then 0.1 M TSC was added dropwise. The mixture solution was subjected to ultrasonication radiation using Hielscher UP400S (400 W, 24 kHz) at an amplitude of 67% and a cycle of 0.6 for 10minutes at a temperature of 100°C, until a colour was obtained, which converted after 2 minutes to a colloidal pink color of gold nanoparticles. Then, the synthesis of gold nano-metal/Carbopol hybrid nano-gel was prepared by a sono-chemical method. Typically, 2g of Carbopol 940 was dissolved in 500 ml of deionized water, then it was added to the prepared gold Nano-metal and sonicated with a propeller device, amplitude 92 and cycle 92% for 15 minutes. Then, 200 ml of triethylamine was added with continuous sonication until pink gel formation.

#### 2.2.3 Characterization.

Finally, we characterized the physicochemical properties of the silver and gold nanogels to assess their wound-healing ability. The microscopic characterization investigated the shape and surface features of the silver and gold nanogels, which were confirmed using a transmission electron microscope (TEM) (Jeol, JEM-2100, high-resolution, Japan) and an atomic force microscope (AFM) (5600LS, Agilent, USA). Identification was accomplished by X-ray diffraction (XRD) with the Bruker D8 Discover to recognize silver and gold nanogel crystals, and sufficient preparation without impurity was obtained from the synthesis procedure. The mean size and zeta potential of AgNPs and AuNPs were detected by dynamic light scattering (DLS; Malvern, UK). These indices aimed to acquire information about the morphology and stability of these nanoparticles by zeta potential and size dispersion.

#### 2.2.4 Creation of excision wound.

The rats were anesthetized by i.p. injection of 80/10 mg/kg. B.W. of ketamine hydrochloride/xylazine hydrochloride. The selected back area of the dorsal region was cleaned and shaved by depilatory cream (Reckitt Benckiser, Inc., USA). A circular area was marked using a 2.5 cm × 2.5 cm coin on the dorsal region of each animal, and then the excision wound was made using a sterile surgical blade, according to Shaygan et al. [19]. Care was taken not to traumatize the wound.

#### 2.2.5 Experimental design.

Wounded rats are divided into six groups (6 each) as follows:

Group 1: Untreated animals with no medication are considered a controlled group.
Group 2: Treated topically once daily with a 10% gel-based nanoformulation of silver in the wound area [8].
Group 3: Treated topically once daily with 10% gel-based nanoformulation of gold in the wound area [8].
Group 4: Treated topically once daily with a 10% gel-based nanoformulation of a (1:1) mixture of silver and gold in the wound area [8].
Group 5: Treated topically once daily with Fucidin R cream and considered a reference drug group.

All treatments continued for 21 days.

#### 2.2.6 Wound healing monitoring and measurements.

Wound areas were assessed and photographed at 1st, 4th, 7th, 14th, and 21st. On the 21st day, entire groups of animals were authenticated, and the skin of the wound area was separated. A portion of the skin from each animal was maintained at 10% formalin for histological examination. while, the other portion was washed with cold saline and preserved at −80°C for the estimation of inflammatory mediators of nitric oxide (NO) and myeloperoxidase (MPO) as well as granulation tissue parameters of hydroxyproline (HPR), hexosamine (HXA), and angiogenic indicator; vascular epithelial growth factor (VEGF) by enzyme-linked immunosorbent (ELISA) assay.

#### 2.2.7 Percentage of wound healing measurement.

Each animal’s wound area in mm^2^ was used to calculate the wound healing percentage on the 0th, 3rd, 7th, 14th, and 21st days following the creation of the wound. The animal was maintained in the typical bending position to measure the wound surface. Then, the wound surface was recorded on translucent paper. Finally, the wound healing % was calculated using the following equation, where N indicates the third, seventh, fourteenth, and twenty-first [**Gad *et al*., 2021**].

$\text{Wound healing}\%\;=\frac{\text{Initial wound surface }-\text{Nth day wound surface}}{\text{initial wound surface }}\text{ X 100}$

#### 2.2.8 Biochemical analysis.

A Determination of granulation tissue parameters:

The levels of hydroxyproline, hexosamine, and vascular endothelial growth factor (VEGF) in skin tissue homogenate were evaluated using Enzyme-linked immunosorbent assay (ELISA) kits supplied by MyBioSource®, Inc., USA, according to the manufacturer`s instructions.

B Determination of inflamatory mediators:

Nitric oxide (NO) was measured colorimetrically using a kit purchased from a bio-diagnostic company (Cairo, Egypt). Myeloperoxidase (MPO) was measured using Enzyme-linked immunosorbent assay (ELISA) kits supplied by MyBioSource®, Inc., USA, according to the manufacturer`s instructions.

#### 2.2.9 Histological examinations.

Tissue samples from the skin wound were kept in a 10% neutral buffered formalin, routinely processed, stained, and examined under a light microscope as described by Abdellatif et al. [20]. Some of the slices of 5 µm were stained with hematoxylin and eosin (H&E); the other sections were further stained with Masson’s trichrome (MT) for investigating the collagen fibers, and histological variations were detected by an optical microscope (Olympus).

#### 2.2.10 Statistical analysis.

In this study, all values were expressed as Mean ± SD of the mean. Data analysis was performed using the SPSS software version19. The difference between groups was assessed using a one-way analysis of variance (ANOVA) at p< 0.05. Consequent multiple comparisons between the different groups were analyzed by Tukey’s test at p< 0.05.

#### 2.2.11 Molecular docking method.

**1 Receptor preparation:**

Two-dimensional x-ray crystal structures (collagenase, elastase, and trypsinase) were obtained from uniport (https://www.uniprot.org/uniprotkb) as a receptor pdb using alpha fold accessions AF_O88766, AF-D4A488, and AF-W4VSR7, respectively, for all in silico molecular docking calculations. Crystal structures were prepared by including all hydrogen, while missing residues were constructed using Autodock Tools 1.5.7 and saved as PDBQT individual files.

**2 Binding site detection:**

The Deep Site Playmolecule tool detected the binding pockets using a neural network deep learning algorithm for the envelope protein. As a result of the ligand-free state of the record, by submitting the PDB ID, three different amino acids were recorded around the highest score pocket (Tyr138, Asp139, and Thr160). For protease proteins, the binding site is determined according to the dimensions of the co-crystalized inhibitor.

**3 ligand preparation:**

Au and Ag were generated, and their energy was minimized using Avogadro 1.2.0 and the Universal Force Field (UFF). Due to the inorganic nature of the ligand, a force field was applied to reduce DeltaE to the zero state of the ligand.

**4 Molecular docking analysis:**

AutoDock 1.5.2 software was used to perform all molecular docking calculations. The PDBQT file for the receptors was generated according to the AutoDock protocol. The parameters for the genetic algorithm number (GA) and the maximum energy evaluations (eval) were set to 250 and 25,000,000, respectively. All other settings remained at their default values. The docking grid dimensions were defined to enclose the active sites. For collagenase: X = 11.15, Y = 7.053, Z = 9.647 (20x20x20), Elastase: X = 4.419, Y = 6.428, Z = −11.25 (20x20x20), and Trypsinase X = 0.379, Y = −3.135, Z = −12.516 (20x20x20). As well, the grid spacing value was set to 0.375 Å. The grid center’s coordinates were positioned, the probable binding modes for each studied analogue were processed by built-in clustering analysis (2.0 Å RMSD tolerance), and the conformation with the lowest energy in the largest cluster was picked out as representative. ADMET investigation: The potential toxicity risks of the compounds (silver, gold, and their combination) depended on their ADMET characteristics. The ADMET evaluations were using ADMET-SAR (https://ai-druglab.smu.edu/admet).

## 3 Results

### 3.1 Results of characterization

**Fig 1:**
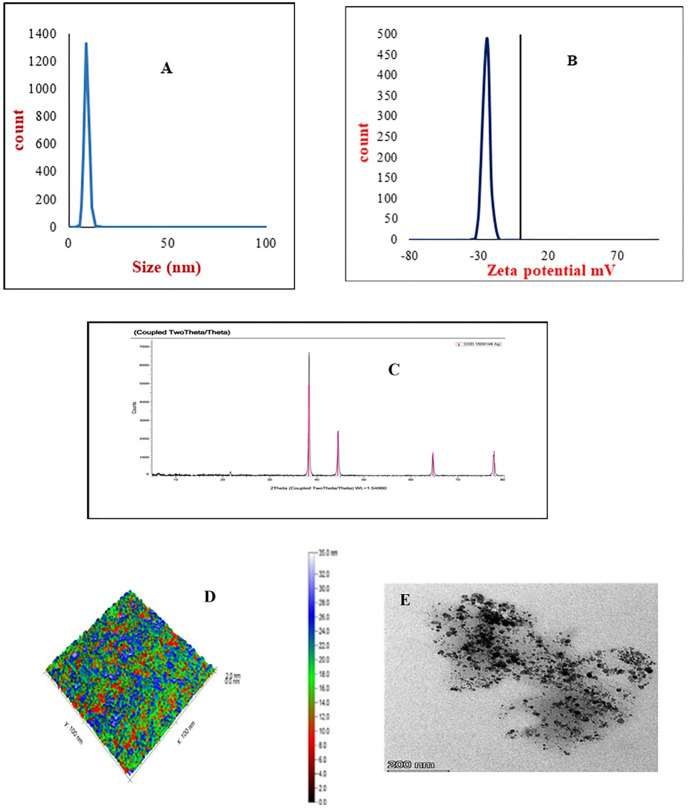
(A) Dynamic light scattering (DLS), (B) Zeta potential, (C) X-ray diffraction (XRD), (D) Atomic force microscopy (AFM), and (E) Transmission electron microscopy (TEM) of silver nanoparticles gel.

**Fig 2:**
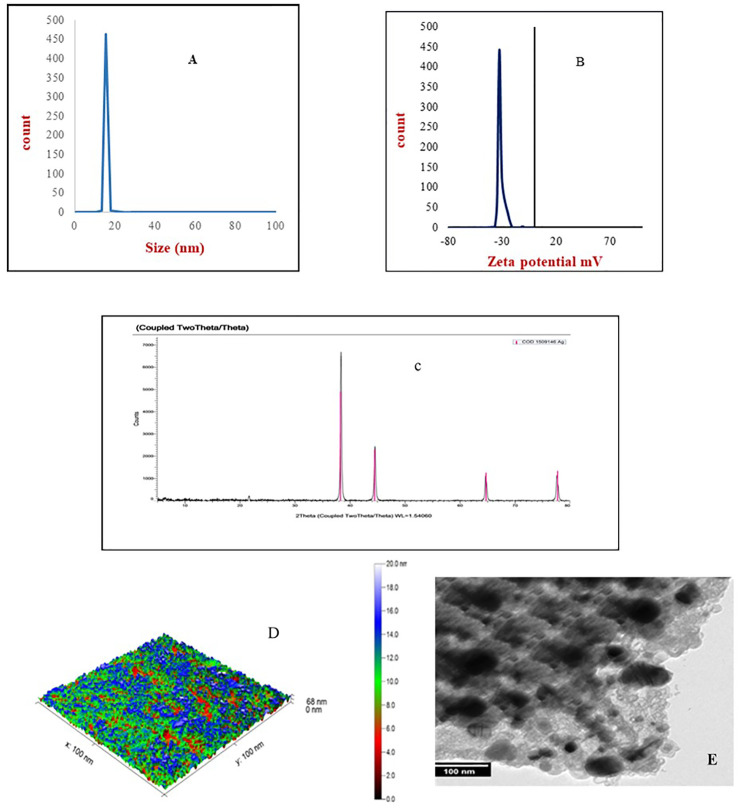
(A) Dynamic light scattering (DLS), (B) Zeta potential, (C) X-ray diffraction (XRD), (D) Atomic force microscopy (AFM), and (E) Transmission electron microscopy (TEM) of gold nanoparticles gel.

Dynamic scattering investigated the particle size and aggregation state of nanoparticle dispersion in solution. Results of the DLS histogram showed that the average particle size (P.S.) and polydispersity index (PDI) were 10 nm, 0.22 (AgNPs), 15 nm, 0.21 (AuNPs), respectively (Figs 1 and 2). The results of average P.S. and PDI indicated that the particle size falls within the nano-scale, with a suitable PDI value reflecting the homogeneity of the nanoparticles’ dispersion. Additionally, zeta potential analysis measured the surface charges of both Ag and Au nanoparticles. The Zeta potential of prepared nanoparticles was −24 mV (AgNPs), -32mV (AuNPs), which referred to AuNPs being more stable than AgNPs in nature (Figs 1 and 2). The XRD pattern of the silver and gold nanoparticles demonstrated the XRD fingerprint pattern for silver and gold nanogel, according to JCPDS file No. 04–0783 and 04–0784, which confirmed the presence of silver or gold ions and crystalline nature of silver or gold nanoparticles, respectively. The gel matrix didn’t show any peaks because of its amorphous nature. Images of TEM and AFM showed the spherical shape of silver and gold nanoparticles at the nanoscale, and dispersed in the gel matrix without aggregation.

### 3.2 Results of % wound contraction area

**Fig 3:**
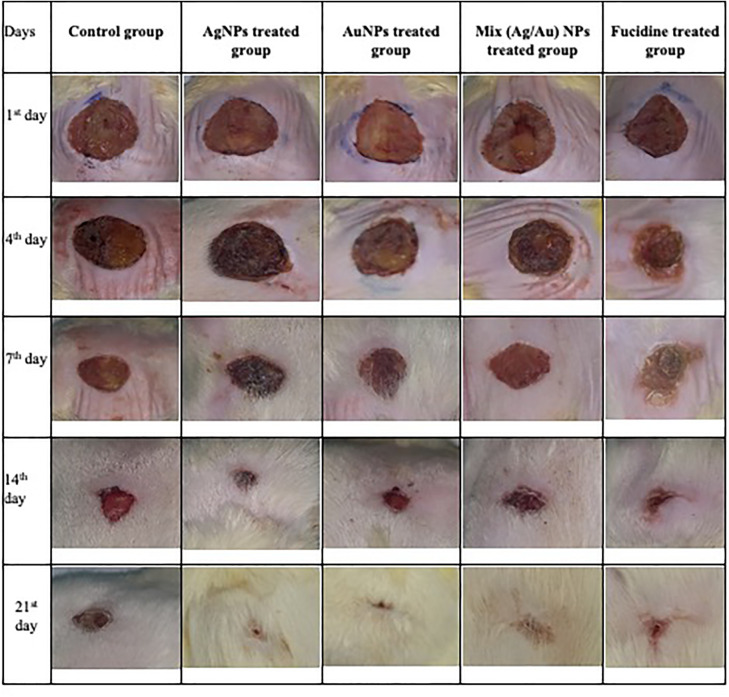
Photographs of the wound healing effect of different treatment groups at different daily intervals (1^st^, 4th, 7^th^, 14^th^, and 21^st^ days).

The initial size of the wound was measured along with the closure progression. The wound contraction rate was evaluated by measuring the reduction in wound size on the 3rd, 7th, 14th, and 21st days after treatment. The enhancement in wound contraction was compared between the excision wounds that were treated and those in the control (untreated) group (Table 1). Among all the treatment groups, those receiving AgNPs, AuNPs, and Fucidin demonstrated superior wound healing properties compared to the Mix group (Fig 3). The percentage of wound contraction observed in the Ag NPs group was the highest, reaching 98 ± 1.7 (almost complete closure) by day 21, followed by the group treated with AuNPs. In contrast, the Mix-treated group exhibited the smallest wound contraction area relative to both the control group and the other treatment groups. The respective figures for the control (untreated) group and the standard antibiotic (Fucidin) were 64.7 ± 2.9 and 95.7 ± 1.7 on the 21st day. The percentage of wound contraction in the AuNPs group was similar to that of Fucidin, at 94.6 and 91.2 percent, respectively (Table 1). The healing rates for the AuNPs and AgNPs groups were significantly quicker than those for the Mix group, and the outcomes from the excision wound healing model were reported as mean ± SD and analyzed by ANOVA (Table 1).

**Table 1:** Effect of silver, gold, and their mixed nanogel formulations on excision wound model (%wound contraction) in female albino rats on 1st, 4th, 7th, 14th, and 21st days.

Groups	Day4 Mean± SD	Day7 Mean± SD	Day14 Mean± SD	Day 21 Mean± SD
**Control**	13.7 ± 0.44^**A**^	32.7 ± 1.4^**A**^	42.1 ± 1.3^**A**^	64.7 ± 2.9^**A**^
**AgNPs**	45.2 ± 3.1^**E**^	71.76 ± 2.2^**E**^	88.2 ± 2.7^**E**^	98.0 ± 1.7^**D**^
**AuNPs**	34.3 ± 2.1^**D**^	65.8 ± 3.4^**D**^	81.9 ± 1.5^**D**^	94.6 ± 2.4 CD
**Mix**	22.3 ± 1.8^**B**^	41.8 ± 1.8^**B**^	52.9 ± 2.3^**B**^	76.6 ± 2.6^**B**^
**Fucidin**	27.2 ± 1.8^**C**^	54.18 ± 2.7^**C**^	74.9 ± 3.2^**C**^	91.2 ± 0.94^**C**^

Values were expressed as mean ±SD(n = 6), In the same column, the same superscript letters indicated non-significant difference and different superscript letters indicated the significant difference between the different groups. ANOVA test followed by turkey’s multiple comparison test between groups at P < 0.05 were applied.

### 3.3 The effect of different nanogel formulations on inflammatory mediators

**Fig 4:**
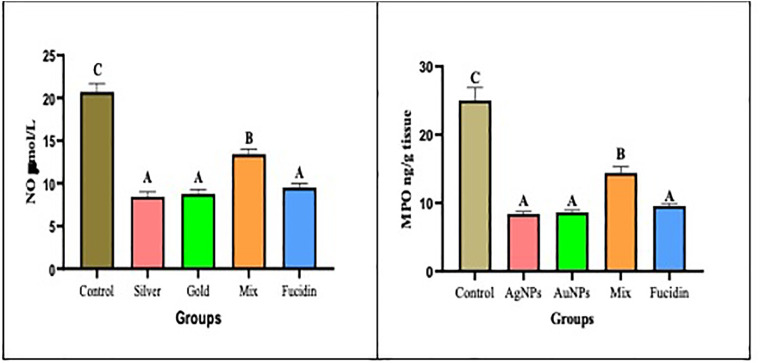
Showing the effect of different nanogel formulations on inflammatory mediators (NO and MPO, respectively) on the 21^st^ day. Values were expressed as mean ±SD(n = 6), In the same column, the same superscript letters indicated non-significant difference and different superscript letters indicated the significant difference between the different groups. ANOVA test followed by turkey’s multiple comparison test between groups at P < 0.05 were applied.

Fig 4 illustrates the effects of various treatments involving AgNPs, AuNPs, a combination of AgNPs/AuNPs, and Fucidin cream on two inflammatory mediators, namely MPO and NO content, during the excision wound healing process. Initially, there was a significant decrease in both MPO content and NO in the groups treated with AgNPs, AuNPs, and Fucidin compared to the untreated control group (p < 0.05). However, there were no significant differences among these treated groups, which is associated with the reduction in ROS production due to AgNPs and AuNPs administration. Additionally, a potential increase was observed in the mixed treatment groups when compared to the non-treated control groups (p < 0.05).

### 3.4 The effect of different nanogel formulations on vascular endothelial growth factor

**Fig 5:**
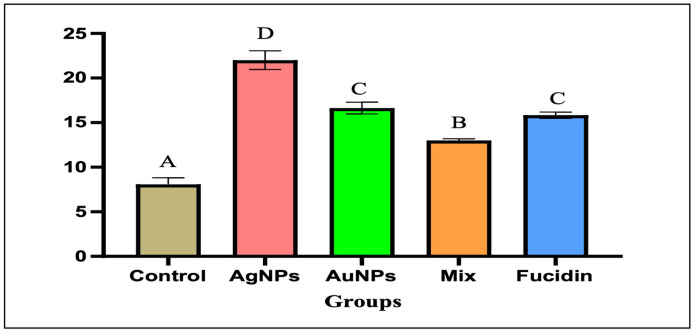
Showing the effect of different nanogel formulations on the angiogenic factor (VEGF) on the 21^st^ day. Values were expressed as mean ±SD(n = 6), In the same column, the same superscript letters indicated non-significant difference and different superscript letters indicated the significant difference between the different groups. ANOVA test followed by turkey’s multiple comparison test between groups at P < 0.05 were applied.

VEGF is the most vital and dominant long-term enhancer of angiogenesis in wound sites. On day 21, VEGF levels in the wound were measured in all treated groups using the ELISA technique (Fig 5). Both the AgNPs and AuNPs groups exhibited higher levels compared to the control group or their combination (P < 0.05). Conversely, there were no significant differences observed in VEGF levels between the AgNPs and Fucidin acid-treated groups.

### 3.5 The effect of different nanogel formulations on granulation tissue contents

**Fig 6:**
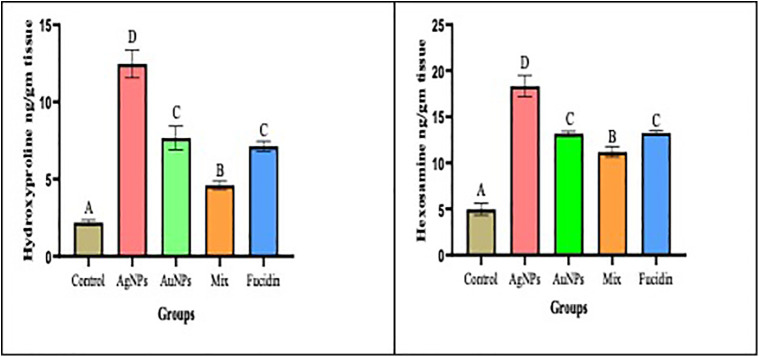
Showing the effect of different nanogel formulations on granulation tissue contents (hydroxyproline and hexosamine, respectively) the 21^st^ day.

Fig 6 illustrates the concentrations of hydroxyproline and hexosamine found in skin tissues. All treated groups showed a significant increase in both hydroxyproline and hexosamine levels. The hydroxyproline and hexosamine contents were higher in the AgNPs group compared to the other treated groups, followed by the AuNPs group. There was no significant difference between the AuNPs and the Fucidin-treated group. However, throughout the healing process, the mixed group showed the lowest levels of hydroxyproline and hexosamine compared to the other treated groups.

Values were expressed as mean ±SD(n = 6), In the same column, the same superscript letters indicated non-significant difference and different superscript letters indicated the significant difference between the different groups. ANOVA test followed by turkey’s multiple comparison test between groups at P < 0.05 were applied.

### 3.6 Results of histological variations

**Fig 7:**
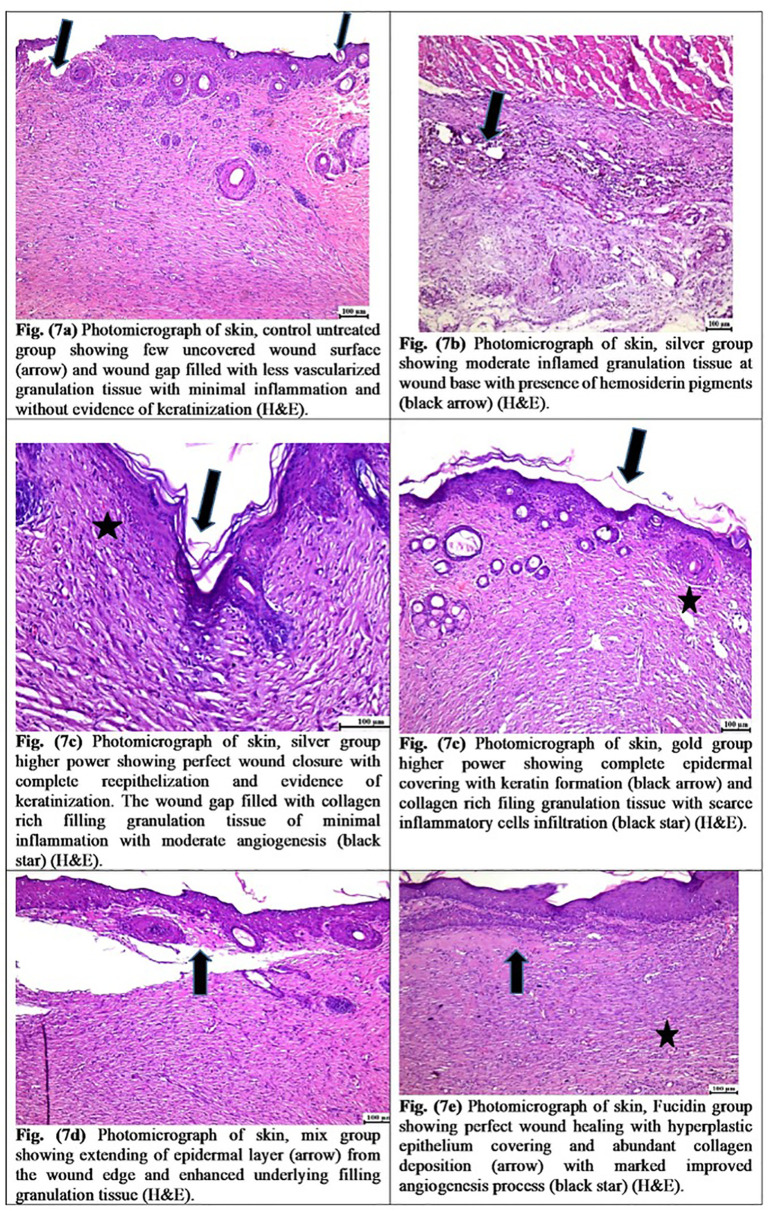
Showing the effect of different nanogel formulations on histological alterations on the 21^st^day.

Microscopic examination of the wound area in the control untreated group (Fig 7a) revealed fewer signs of delayed healing; the wound surface showed most epidermal cover of the wound surface without evidence of keratinization, and the granulation tissue that filled the wound gap contained varying numbers of inflammatory cells and collagen. There was infiltration with fewer newly formed blood capillaries.

Regarding the silver group (Fig 7b), there was a complete healthy layer of stratified squamous keratinizing epithelium covering the wound surface. In several examined sections, granulation tissue with collagen deposition filled the wound gap. Some examined sections showed an increased number of inflammatory cells infiltration with the presence of golden yellow to brown hemosiderin pigment.

In the gold group (Fig 7c), a higher degree of wound healing was observed, characterized by complete epidermal remodeling with an existing keratin cover. The wound gap was occupied by well-vascularized granulation tissue with extensive collagen deposition.

Mixed groups showed poor wound healing (Fig 7d). The injured surface showed a partial epidermal layer, limited to the edge and lacking in the center of the wound. The wound gap showed superficial poor-quality granulation tissue in the exposed area with increased infiltration of inflammatory cells. However, the base of the wound and the covered wound surface showed enhanced collagen deposition in the formed granulation tissue, a minimal inflammatory reaction with enhanced angiogenesis.

In the Fucidin group, a high rate of wound healing was shown (Fig 7e). A hyperplastic keratinized layer covered the wound surface. High-quality vascularized granulation tissue and abundant collagen deposition filled the wound gap.

In comparison to the control non-treated group, the silver, gold, and Fucidin-treated groups showed significant improvement in re-epithelization, angiogenesis, and granulation tissue scores. Although in the inflammatory score, no significant difference was recorded between the silver, gold, and Fucidin treated groups. The only mixed group showed a significant difference when compared to the other treatment groups and the control, untreated group.

**Fig 8:**
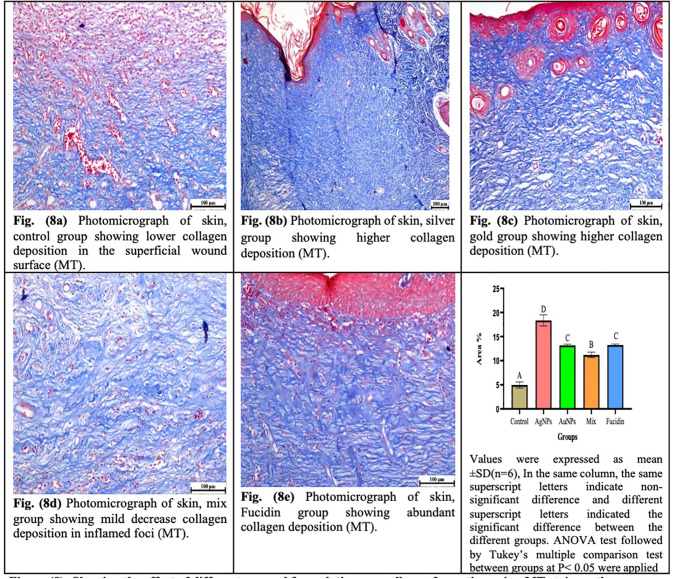
Showing the effect of different nanogel formulations on collagen formation using MT stain on the 21^st^day.

Outcomes of the tissue biopsies’ MT staining on the 21st day of wound healing are displayed in Fig 8. The maximum amount of collagen bundle renewal was detected in treated AgNPs, followed by AuNPs or Fucidin-treated groups with respect to control-handled groups or the mixed treated groups. There was no significant difference between AuNPs and Fucidin-treated groups with respect to the control-handling group (P < 0.05).

### 3.7 Molecular docking analysis

**Fig 9:**
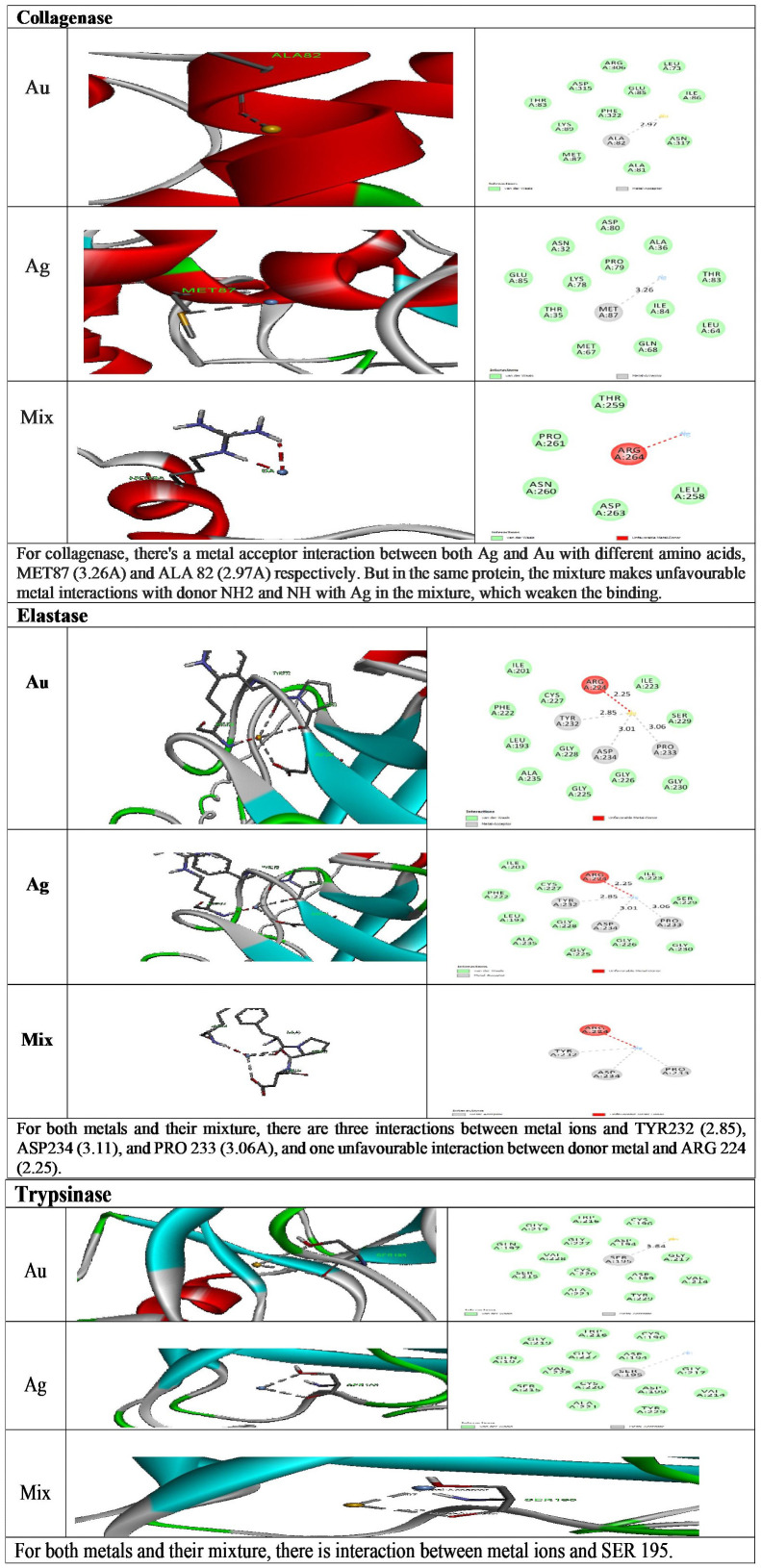
Showing the molecular docking of metal ions (Au, Ag and their mixture) with protease enzymes (collagenase, elastase and trypsinase).

The outcomes of the molecular docking investigation of the examined compounds as prospective inhibitors of protease enzymes involved their corresponding areas and scores using PatchDock (Table 2) and binding free energy, their inhibition constant (Ki), and their respective interacting residues using Autodock (Table 3 and Fig 9). The obtained scores and areas using PatchDock analysis, we can see that the values for the individual metal-protein complexes (Ag and Au) are the same, indicating that the metals do not have a significant effect on the binding affinity of the proteins. Nevertheless, we found that the binding affinity of collagenase decreased when the metals were mixed, although the binding affinities of trypsinase and elastase significantly increased. Results of Autodock analysis showed that the Au molecule exhibited the best binding affinity with the collagenase molecule, followed by the mixture of Au and Ag; however, the Ag molecule had the weakest binding affinity. For elastase, based on these results, both the Ag and Au molecules had similar binding affinities with elastase, and the mixture of the two metals did not show any significant changes in binding affinity. The values of the molecular docking simulations of Au, Ag, and a mixture of these metals with trypsinase provided information about the potential binding affinity of these metals with the enzyme. Both Au and Ag metals exhibited a moderately strong binding affinity with trypsinase. However, the binding affinity between trypsinase and the mixture of Au and Ag is weaker than with either metal alone.

**Table 2:** Showing patch Dock results of Au, Ag nanoparticles, or their mixture on protease enzymes (collagenase, elastase, and trypsinase).

Examined elements	collagenase		Elastase		Trypsinase	
	Score	Area	Score	Area	Score	Area
Au	460	58.5	404	55.4	390	59.6
Ag	460	58.5	404	55.4	390	59.6
Mix	404	55.4	892	119.8	790	96.2

The score indicates the binding affinity between the metal-protein complexes, while the areas correspond to the size of the binding interface between them

**Table 3:** Showing Autodock results of Au, Ag nanoparticles, or their mixture on protease enzymes (collagenase, elastase, and trypsinase).

Examined elements	collagenase			Elastase			Trypsinase		
	Ki	Free binding energy	Interacting amino acids residues	Ki	Free binding energy	Interacting amino acids residues	Ki	Free binding energy	Interacting amino acids residues
**Au**	886.02 mM	−0.07 kcal/mol	MET87	811.57 mM	−0.12 kcal/mol	TYR232 ASP234 and PRO 233	765.02 mM	−0.16 kcal/mol	SER 195
**Ag**	985.44 mM.	−0.01 kcal/mol	ALA 82	811.57 mM	−0.09 kcal/mol	TYR232 ASP234 and PRO 233	800.69 mM	−0.13 kcal/mol	SER 195
**Mix**	922.65 mM	−0.05 kcal/mol	unfavourable interactions with donor NH2 and NH	811.57 mM	−0.09 kcal/mol	TYR232 ASP234 and PRO 233	Non estimated	+0.17 kcal/mol	SER 195

### 3.8 The prediction of the ADMET properties of the examined nanometals

The ADMET properties of a specific molecule play a crucial role in determining the efficacy, safety, and pharmacokinetic profile of a metal [21], as shown in Table 4.

**Table 4:** Showing the forecasting ADMET properties of Au, Ag, or their mixture.

Molecule Property	Value) Ag)	Value (Au)	Value (Ag/Au mix)	Unit	Range
**Molecular Weight**	106.91	196.97	303.87	kg/mol	100-600
**Number of heteroatoms**	1	1	2	/	1-15
**Number of Rotatable Bonds**	0	0	0	/	<=11
**Number of Rings**	0	0	0	/	<=6
**Number of hydrogen bond acceptor (HBA)**	0	0	0	/	<=12
**Number of hydrogen bond donor (HBD)**	0	0	0	/	<=7
**n-octanol/water partition coefficient (K**_**OW)**_	−0.0	−0.0	−0.01	log-ratio	0-3
**Absorption**	**Prediction**	**Prediction**	**Prediction**	**Unit**	
**Caco-2 cell permeability**	−4.44	−4.48	−4.29	log(cm/s)	>-5.15
**Human intestinal absorption (HIA)**	68.75	68.31	49.05	%	>=80%
**P glycoprotein inhibition (Pgp Inhibition)**	10.68	9.5	13.52	%	<=30%
**Logarithm of the distribution at constant PH (log D**_**7.4**_ **)**	0.68	0.69	0.53	log-ratio	1-3
**Aqueous Solubility**	−1.78	−3.54	−3.47	log(mol/L)	Slightly soluble
**Oral Bioavailability**	70.29	68.16	62.91	%	Moderately high
**Distribution**	**Prediction**	**Prediction**	**Prediction**	**Unit**	
**Blood brain barrier (BBB)**	32.36	64.14	52.03	%	<=30%
**Plasma protein binding rate** (**PPBR)**	45.58	44.08	38.17	%	<=90%
**Volume of distribution at steady state (VDss)**	2.66	2.78	2.78	L/kg	0.02-20
**Metabolism**	**Prediction**	**Prediction**	**Prediction**	**Unit**	
**CYP2C9 Inhibition**	17.32	17.49	11.98	%	Low
**CYP2D6 Inhibition**	10.92	9.9	22.81	%	Low
**CYP3A4 Inhibition**	20.67	21.9	26.37	%	Low
**CYP2C9 Substrate**	19.96	21.08	21.91	%	Low
**CYP2D6 Substrate**	33.01	28.87	40.57	%	Low
**CYP3A4 Substrate**	27.75	26.57	28.92	%	low
**Excretion**	**Prediction**	**Prediction**	**Prediction**	**Unit**	
**Half-life (t½)**	5.27	6.73	6.26	hr.	Low
**Hepatic clearance (CL-Hepa)**	36.22	32.01	28.87	µL min^-1^ (10^6^ cells)^-1^	Low
**Microsomal clearance (CL-Micro)**	23.49	22.49	31.27	mL min^-1^ g^-1^	Low
**Toxicity**	**Prediction**	**Prediction**	**Prediction**	**Unit**	
**Human ether-a-go-go related gene (hERG) blockers**	19.31	18.51	24.04	%	<=30%
**Ames test**	41.93	41.44	39.3	%	<=30%
**Drug induced liver injury (DILI)**	21.98	18.37	21.33	%	<=30%
**Lethal dose 50%** **(LD50)**	1.44	1.33	1.66	-log(mol/kg)	

#### 3.8.1 Molecular weight.

According to reports, the molecule recorded a molecular weight of 106.91 kg/mol, which was in the typical range of 100–600 kg/mol for organic molecules. Several aspects of solubility, permeability, and distribution within the body were affected by molecular weight.

#### 3.8.2 Absorption.

The Caco-2 permeability of the molecule indicated its ability to pass through the intestinal epithelium. The recorded values ranged from −4.44 to −4.29 log (cm/s), suggesting moderate to high permeability, which implied that the molecule had the potential to be well absorbed through the gastrointestinal tract if taken orally.

#### 3.8.3 Oral bioavailability.

The molecule’s oral bioavailability ranged from 62.91% to 70.29%. These values indicated that a significant proportion of the administered dose could reach the systemic circulation, suggesting favorable oral absorption.

#### 3.8.4 Distribution.

The distribution of molecules throughout the body was estimated by considering the percentages of blood-brain barrier (BBB) and plasma protein binding (PPBR). The BBB values ranged from 32.36% to 64.14%, indicating the molecule’s ability to cross the blood-brain barrier to varying degrees. The PPBR values ranged from 38.17% to 45.58%, suggesting moderate to high plasma protein binding.

#### 3.8.5 Metabolism.

The predicted molecule’s metabolism was evaluated based on their interactions with specific cytochrome P450 enzymes (CYP2C9, CYP2D6, and CYP3A4). The data indicated both inhibitory and substrate characteristics for these enzymes. These findings imply that the molecule might undergo metabolic transformations and interact with drug-metabolizing enzymes in the body.

#### 3.8.6 Excretion.

The predicted half-life of the molecule ranged from 5.27 to 6.73 hrs., suggesting moderate persistence within the body. The clearance rates (CL-Hepa and CL-Micro) indicated the elimination of the molecule from the body, with values ranging from 28.87 to 36.22 µL min-1 (106 cells)-1 and 22.49 to 31.27 mL min-1 g-1, respectively.

#### 3.8.7 Toxicity.

The molecule’s toxicity was evaluated based on various factors such as hERG blockers, the Ames test, and DILI (drug-induced liver injury) potential. The results fell within acceptable limits, with percentages ranging from 18.37% to 41.93% for different toxicity assays, suggesting a relatively low risk of adverse effects.

## 4 Discussion

Nanoformulations have developed a technique to synthesize pure gold and silver nanoparticles; this technique increased the release rate of gold and silver ions ([22], **Ramadan *et al.*, 2018]**.

These results revealed that the mean sizes of the AgNPs and AuNPs were 10 nm and 15 nm, respectively. Our findings aligned with those of **Pivodova *et al.* (2015)**, who observed that gold nanoparticles (AuNPs) larger than 4–5 nm did not exhibit toxicity after short-term exposure. But gold nanoparticles smaller than 4 nm have converted into catalytically active forms and became cytotoxic when measured in zebrafish models. Similarly, Mihai et al. [15] reported that new silver nanoparticle commercial dressings have been implemented in various clinical trials for wound healing, particularly for burns and chronic wounds, such as Acticoat, which has an average particle size of 15 nm.

Typically, Electrostatic stabilization relies on the formation of a charged layer from the adsorption of ionic groups on the nanoparticle surface, which prevents particle agglomeration through their repulsive forces. According to this principle, the Zeta potential of the synthesized nanoparticles was −24 mV for AgNPs and −32 mV for AuNPs, indicating that gold nanoparticles are relatively more stable in their natural state. The DLS and zeta potential results for the prepared Ag and Au nanoparticles were consistent with those of Gaikwad et al. [23]. Transmission electron microscopy and atomic force microscopy images revealed that silver and gold nanoparticles exhibited a uniform spherical shape without agglomeration, and homogeneous distribution within the gel matrix.

The rate of wound size reduction was the actual assessment of the wound healing measurement. Subsequently, AgNPs and AuNPs enhanced wound healing compared to standard drugs or when mixed with them. Fibroblast growth and collagen fiber deposition resulted in a synchronized reduction in the wound area and surface. Furthermore, the formulated nanogels have enhanced the period of epithelization. Consequently, these findings demonstrated that AgNPs and AuNPs effectively enhanced the wound-healing process, which aligned with the results obtained by Shanmugasundaram et al. [8], Dwivedi et al., [24] **and** Toczek et al., [25].

The inflammatory response is a vital component of wound healing, aiming to stop the growth of pathogens in the wounded area, inhibit infection, subsequently increase the number of fibroblast cells, and promote collagen formation. Although these factors are essential for starting, maintaining, and controlling the post-injury response, they have also contributed to slow wound healing and defects in scar formation. Fewer neutrophils and macrophages are attracted to the wound, and fewer mediators are discharged into the wound, resulting in lower cellular proliferation, fibroblast and keratinocyte relocation, and ECM formation. Typically, downregulation of NO production delays wound healing by decreasing collagen deposition and reducing wound tensile strength. Sustained high concentrations of NO may needlessly prolong the inflammatory phase of wound healing, leading to keloid formation [26,2]. So, the ratios of pro-inflammatory and anti-inflammatory mediators would be properly balanced during effective wound healing.

Moreover, the Myeloperoxidase (MPO) enzyme, a heme-containing protein released by neutrophils, acts as an initial indicator of inflammation or cell and tissue injury. Additionally, previous research indicated that MPO levels were significantly lower in wound fluid (WF) from acute wounds than those in the tissues of pressure ulcers. Also, MPO has been effectively used to monitor a specific wound therapy [7**].** In this context, Rajendran et al. [27] reported that AgNPs exhibited anti-inflammatory activity through enhancing neutrophil apoptosis. As a result, low levels of pro-inflammatory mediators accelerated wound remodeling and reduced hypertrophic scarring. **Ramadan *et al.* (2018)** found that the levels of pro-inflammatory mediator IL-6 mRNA were significantly lower during the early stages of repair in wounds treated with silver nanoparticles. Our findings regarding inflammatory mediators, such as nitric oxide (NO) and myeloperoxidase (MPO) levels, indicated that silver nanoparticles (AgNPs) and gold nanoparticles (AuNPs) demonstrated significant anti-inflammatory effects, reduced MPO levels, alleviated inflammation and oxidative damage, and enhanced the healing process [28].

During angiogenesis, the wound site becomes abundant in blood vessels, which is necessary for wound nutrition. Therefore, the factor that stimulates angiogenesis can cause the normal development of the healing process. These key factors included in wound remodeling were VEGF and collagen molecules [29]. Our results showed a significant difference in VEGF activity and collagen density, with superior values in the managed groups, particularly in the AgNPs nanoparticle treatment group, followed by AuNPs, compared with the standard managed and control groups, which enhanced angiogenesis and wound closure. Our results were consistent with those of Ramadan et al. [30], who found that during wound healing in mice, VEGF mRNA levels were higher in the AgNPs-treated group than in the standard-treated group. The granulation tissue in the wound area was suppressed by the AntiVEGF, demonstrating role of VEGF in angiogenesis during the proliferative phase. Likewise, earlier research by Patel et al. [31] **and** Yubiao Liu et al. [29] reported that VEGF activity had reduced in diabetic wounds, and resulting in delayed wound remodeling.

Subsequently, collagen plays an essential role in wound healing. Hexosamine and hydroxyproline levels in healing wounds after 21 days were estimated in our study, and the groups treated with AgNPs and AuNPs had a considerably higher hexosamine content than the control or mix groups.

In contrast, the mix group level of hydroxyproline and hexosamine were noticeably lower than those of the control untreated group. According to Dwivedi et al. [24], the tensile strength of wounds couldn’t heal without the synthesis of collagenous components such as hydroxyproline and hexosamine. A higher hydroxyproline indicate a quicker rate of the wound remodeling. Augmented hydroxyproline content, which is a reflection of augmented cellular proliferation and subsequently amplified collagen production. A significant level of hexosamine indicates the stabilization of collagen moieties by stimulating electrostatic and ionic interfaces. Also, Shanmugasundaram et al. [8] discovered that wound repair at different daily intervals after management had significant amounts of hexosamine, hydroxyproline and uronic acid. According to our research, AgNPs and AuNPs significantly enhanced collagen synthesis and cellular proliferation in the excision wound area, demonstrating their ability to repair wounds. Burn management with silver sulfadiazine has been used for several years. However, poor absorption might be the cause of pure silver’s lower benefits **[Konob *et al*., 2016]**. So, we handled the pure AgNPs and AuNPs, which improved wound healing.

The histochemical and MT staining findings showed that wound healing rates, as well as tissue regeneration, were significantly correlated with greater production of hydroxyproline, hexosamine, deposition of collagen fibers, and lower activities of both NO and MPO in skin wounds treated with the AgNPs and AuNPs than in control non-treated wounds or their mix treated group.

Results of molecular docking provided valuable insights into how different metals can affect the binding affinity of these proteins and significantly impact the inflammatory response. Inflammation is a multifaceted biological process that activates various proteins, including enzymes such as collagenase, elastase, and trypsinase. The interaction of these proteins with metal ions might control the inflammatory response. These could be crucial for the development of metal-based treatments for inflammatory conditions such as arthritis and inflammatory bowel syndrome, as the pathophysiology of these disorders includes these particular enzymes [32]. If the binding affinity is too weak, the drug may not effectively target the protein. Therefore, it may be ineffective in treating the underlying inflammatory condition, as observed in the interaction of the metal mixture with collagenase and trypsinase compared to either metal alone. Overall, these analyses provide valuable information for checking the potential role of metals in modulating the inflammatory response. Further research is needed to fully explore the implications of these findings for the development of metal-based therapeutics for inflammatory conditions [33].

## 5 Limitations

A key limitation of this study was the lack of a power analysis to determine the appropriate sample size for the recent study. Ultimately, the topical applications of formulated gold and silver nanogels demonstrated greater effectiveness in reducing inflammation and promoting re-epithelialization compared to the mixed group during the repair process. The mechanism behind the continuous repair action of both types of formulated nanogels may be their angiogenic and anti-inflammatory properties according to Naraginti, S., et al. [34] **and** Naskar and Kim [35].

## 6 Conclusion

The *in vivo* study of metal nanoformulations for wound healing using the excision model demonstrated that gels containing AgNP or AuNP had superior wound-healing effects compared to their mixtures or control groups. The anticipated role of nanoparticles in wound healing may be due to their ability to decrease free radicals and myeloperoxidase-generated tissue damage, reduce inflammation, and accelerate collagen deposition, as shown by histopathological evidence and smaller wound area percentages. Furthermore, *in silico* results showed that AgNPs or AuNPs are more effective in inhibiting protease enzymes involved in inflammatory responses. *In silico* and *in vivo* results indicated that silver or gold nanogels could be promising for use in wound healing dressings.

## Supporting information

S1 File Supporting information and row data. (DOCX)
