# Supplementary material for: Comparative evaluation of the healing potential of excision wound using Nano- formulations of silver, gold, or their mixture: In-silico and in vivo approaches
Source: PLoS One. 2026 Apr 30;21(4):e0347682. doi: 10.1371/journal.pone.0347682 (PMC13132192; doi:10.1371/journal.pone.0347682)
Supplement: S1 File — (DOCX) [file pone.0347682.s001.docx]

**Descriptive analysis and multiple comparison of figure (4) of NO level at P<0.0001**

| Mean | 20.71 | 8.450 | 8.750 | 13.43 | 9.500 |
| --- | --- | --- | --- | --- | --- |
| Std. Deviation | 0.9869 | 0.5568 | 0.5568 | 0.5909 | 0.4761 |
| Std. Error of Mean | 0.4934 | 0.2784 | 0.2784 | 0.2955 | 0.2380 |
|  |  |  |  |  |  |
| Lower 95% CI | 19.14 | 7.564 | 7.864 | 12.48 | 8.742 |
| Upper 95% CI | 22.28 | 9.336 | 9.636 | 14.37 | 10.26 |

| Tukey's multiple comparisons test | Mean diff. | 95.00% CI of diff. | Below threshold? | Summary | Adjusted P Value |  |
| --- | --- | --- | --- | --- | --- | --- |
| Control vs. Silver | 12.26 | 10.82 to 13.70 | Yes | **** | <0.0001 | A-B |
| Control vs. Gold | 11.96 | 10.52 to 13.40 | Yes | **** | <0.0001 | A-C |
| Control vs. Mix | 7.288 | 5.849 to 8.726 | Yes | **** | <0.0001 | A-D |
| Control vs. Fucidin | 11.21 | 9.774 to 12.65 | Yes | **** | <0.0001 | A-E |
| Silver vs. Gold | -0.3000 | -1.738 to 1.138 | No | ns | 0.9651 | B-C |
| Silver vs. Mix | -4.975 | -6.413 to -3.537 | Yes | **** | <0.0001 | B-D |
| Silver vs. Fucidin | -1.050 | -2.488 to 0.3884 | No | ns | 0.2133 | B-E |
| Gold vs. Mix | -4.675 | -6.113 to -3.237 | Yes | **** | <0.0001 | C-D |
| Gold vs. Fucidin | -0.7500 | -2.188 to 0.6884 | No | ns | 0.5135 | C-E |
| Mix vs. Fucidin | 3.925 | 2.487 to 5.363 | Yes | **** | <0.0001 | D-E |

**Descriptive analysis and multiple comparison of figure (4) of MPO level at P<0.0001**

| Mean | 25.03 | 8.375 | 8.605 | 14.42 | 9.550 |
| --- | --- | --- | --- | --- | --- |
| Std. Deviation | 1.852 | 0.3500 | 0.3288 | 0.8959 | 0.3317 |
| Std. Error of Mean | 0.9259 | 0.1750 | 0.1644 | 0.4479 | 0.1658 |
|  |  |  |  |  |  |
| Lower 95% CI | 22.08 | 7.818 | 8.082 | 12.99 | 9.022 |
| Upper 95% CI | 27.97 | 8.932 | 9.128 | 15.85 | 10.08 |

| Tukey's multiple comparisons test | Mean diff. | 95.00% CI of diff. | Below threshold | Summary | Adjusted P Value |  |
| --- | --- | --- | --- | --- | --- | --- |
| Control vs. AgNPs | 16.65 | 14.56 to 18.74 | Yes | **** | <0.0001 | A-B |
| Control vs. AuNPs | 16.42 | 14.33 to 18.51 | Yes | **** | <0.0001 | A-C |
| Control vs. Mix | 10.61 | 8.517 to 12.69 | Yes | **** | <0.0001 | A-D |
| Control vs. Fucidin | 15.48 | 13.39 to 17.56 | Yes | **** | <0.0001 | A-E |
| AgNPs vs. AuNPs | -0.2300 | -2.318 to 1.858 | No | ns | 0.9968 | B-C |
| AgNPs vs. Mix | -6.045 | -8.133 to -3.957 | Yes | **** | <0.0001 | B-D |
| AgNPs vs. Fucidin | -1.175 | -3.263 to 0.9130 | No | ns | 0.4421 | B-E |
| AuNPs vs. Mix | -5.815 | -7.903 to -3.727 | Yes | **** | <0.0001 | C-D |
| AuNPs vs. Fucidin | -0.9450 | -3.033 to 1.143 | No | ns | 0.6383 | C-E |
| Mix vs. Fucidin | 4.870 | 2.782 to 6.958 | Yes | **** | <0.0001 | D-E |

| Tukey's multiple comparisons test | Mean diff. | 95.00% CI of diff. | Below threshold? | Summary | Adjusted P Value |  |
| --- | --- | --- | --- | --- | --- | --- |
| Control vs. AgNPs | -13.92 | -15.71 to -12.12 | Yes | **** | <0.0001 | A-B |
| Control vs. AuNPs | -8.550 | -10.34 to -6.756 | Yes | **** | <0.0001 | A-C |
| Control vs. Mix | -4.917 | -6.710 to -3.123 | Yes | **** | <0.0001 | A-D |
| Control vs. Fucidin | -7.750 | -9.544 to -5.956 | Yes | **** | <0.0001 | A-E |
| AgNPs vs. AuNPs | 5.367 | 3.573 to 7.160 | Yes | **** | <0.0001 | B-C |
| AgNPs vs. Mix | 9.000 | 7.206 to 10.79 | Yes | **** | <0.0001 | B-D |
| AgNPs vs. Fucidin | 6.167 | 4.373 to 7.960 | Yes | **** | <0.0001 | B-E |
| AuNPs vs. Mix | 3.633 | 1.840 to 5.427 | Yes | *** | 0.0004 | C-D |
| AuNPs vs. Fucidin | 0.8000 | -0.9936 to 2.594 | No | ns | 0.6027 | C-E |
| Mix vs. Fucidin | -2.833 | -4.627 to -1.040 | Yes | ** | 0.0029 | D-E |

**Descriptive analysis and multiple comparison of figure (5) of VEGF level at P<0.000**

| Mean | 8.083 | 22.00 | 16.63 | 13.00 | 15.83 |
| --- | --- | --- | --- | --- | --- |
| Std. Deviation | 0.7286 | 1.054 | 0.6506 | 0.2000 | 0.3512 |
| Std. Error of Mean | 0.4206 | 0.6083 | 0.3756 | 0.1155 | 0.2028 |
| Lower 95% CI | 6.273 | 19.38 | 15.02 | 12.50 | 14.96 |
| Upper 95% CI | 9.893 | 24.62 | 18.25 | 13.50 | 16.71 |

|  |  |  |  |  |  |
| --- | --- | --- | --- | --- | --- |
|  |  |  |  |  |  |
| **Descriptive analysis and multiple comparison of figure (6) of hexosamine level at P<0.0001** |  |  |  |  |  |

| Mean | 4.967 | 18.35 | 13.17 | 11.20 | 13.27 |
| --- | --- | --- | --- | --- | --- |
| Std. Deviation | 0.6506 | 1.150 | 0.2517 | 0.5568 | 0.2082 |
| Std. Error of Mean | 0.3756 | 0.6640 | 0.1453 | 0.3215 | 0.1202 |
| Lower 95% CI | 3.350 | 15.49 | 12.54 | 9.817 | 12.75 |
| Upper 95% CI | 6.583 | 21.21 | 13.79 | 12.58 | 13.78 |

| Tukey's multiple comparisons test | Mean diff. | 95.00% CI of diff. | Below threshold? | Summary | Adjusted P Value |  |
| --- | --- | --- | --- | --- | --- | --- |
| Control vs. AgNPs | -13.38 | -15.15 to -11.62 | Yes | **** | <0.0001 | A-B |
| Control vs. AuNPs | -8.200 | -9.967 to -6.433 | Yes | **** | <0.0001 | A-C |
| Control vs. Mix | -6.233 | -8.001 to -4.466 | Yes | **** | <0.0001 | A-D |
| Control vs. Fucidin | -8.300 | -10.07 to -6.533 | Yes | **** | <0.0001 | A-E |
| AgNPs vs. AuNPs | 5.183 | 3.416 to 6.951 | Yes | **** | <0.0001 | B-C |
| AgNPs vs. Mix | 7.150 | 5.383 to 8.917 | Yes | **** | <0.0001 | B-D |
| AgNPs vs. Fucidin | 5.083 | 3.316 to 6.851 | Yes | **** | <0.0001 | B-E |
| AuNPs vs. Mix | 1.967 | 0.1995 to 3.734 | Yes | * | 0.0281 | C-D |
| AuNPs vs. Fucidin | -0.1000 | -1.867 to 1.667 | No | ns | 0.9997 | C-E |
| Mix vs. Fucidin | -2.067 | -3.834 to -0.2995 | Yes | * | 0.0211 | D-E |

**Descriptive analysis and multiple comparison of figure (6) of hydroxyproline level at P<0.000**

| Mean | 2.193 | 12.47 | 7.667 | 4.600 | 7.133 |
| --- | --- | --- | --- | --- | --- |
| Std. Deviation | 0.1677 | 0.9074 | 0.7605 | 0.2646 | 0.3215 |
| Std. Error of Mean | 0.09684 | 0.5239 | 0.4391 | 0.1528 | 0.1856 |
| Lower 95% CI | 1.777 | 10.21 | 5.777 | 3.943 | 6.335 |
| Upper 95% CI | 2.610 | 14.72 | 9.556 | 5.257 | 7.932 |

| Tukey's multiple comparisons test | Mean diff. | 95.00% CI of diff. | Below threshold? | Summary | Adjusted P Value |  |
| --- | --- | --- | --- | --- | --- | --- |
| Control vs. AgNPs | -10.27 | -11.79 to -8.752 | Yes | **** | <0.0001 | A-B |
| Control vs. AuNPs | -5.473 | -6.995 to -3.952 | Yes | **** | <0.0001 | A-C |
| Control vs. Mix | -2.407 | -3.928 to -0.8850 | Yes | ** | 0.0028 | A-D |
| Control vs. Fucidin | -4.940 | -6.462 to -3.418 | Yes | **** | <0.0001 | A-E |
| AgNPs vs. AuNPs | 4.800 | 3.278 to 6.322 | Yes | **** | <0.0001 | B-C |
| AgNPs vs. Mix | 7.867 | 6.345 to 9.388 | Yes | **** | <0.0001 | B-D |
| AgNPs vs. Fucidin | 5.333 | 3.812 to 6.855 | Yes | **** | <0.0001 | B-E |
| AuNPs vs. Mix | 3.067 | 1.545 to 4.588 | Yes | *** | 0.0004 | C-D |
| AuNPs vs. Fucidin | 0.5333 | -0.9883 to 2.055 | No | ns | 0.7760 | C-E |
| Mix vs. Fucidin | -2.533 | -4.055 to -1.012 | Yes | ** | 0.0019 | D-E |

**Descriptive analysis and multiple comparison of figure (8) of area % of MT stain at P<0.0001**

| Mean | 4.967 | 18.35 | 13.17 | 11.20 | 13.27 |
| --- | --- | --- | --- | --- | --- |
| Std. Deviation | 0.6506 | 1.150 | 0.2517 | 0.5568 | 0.2082 |
| Std. Error of Mean | 0.3756 | 0.6640 | 0.1453 | 0.3215 | 0.1202 |
| Lower 95% CI | 3.350 | 15.49 | 12.54 | 9.817 | 12.75 |
| Upper 95% CI | 6.583 | 21.21 | 13.79 | 12.58 | 13.78 |

| Tukey's multiple comparisons test | Mean diff. | 95.00% CI of diff. | Below threshold? | Summary | Adjusted P Value |  |
| --- | --- | --- | --- | --- | --- | --- |
| Control vs. AgNPs | -13.38 | -15.15 to -11.62 | Yes | **** | <0.0001 | A-B |
| Control vs. AuNPs | -8.200 | -9.967 to -6.433 | Yes | **** | <0.0001 | A-C |
| Control vs. Mix | -6.233 | -8.001 to -4.466 | Yes | **** | <0.0001 | A-D |
| Control vs. Fucidin | -8.300 | -10.07 to -6.533 | Yes | **** | <0.0001 | A-E |
| AgNPs vs. AuNPs | 5.183 | 3.416 to 6.951 | Yes | **** | <0.0001 | B-C |
| AgNPs vs. Mix | 7.150 | 5.383 to 8.917 | Yes | **** | <0.0001 | B-D |
| AgNPs vs. Fucidin | 5.083 | 3.316 to 6.851 | Yes | **** | <0.0001 | B-E |
| AuNPs vs. Mix | 1.967 | 0.1995 to 3.734 | Yes | * | 0.0281 | C-D |
| AuNPs vs. Fucidin | -0.1000 | -1.867 to 1.667 | No | ns | 0.9997 | C-E |
| Mix vs. Fucidin | -2.067 | -3.834 to -0.2995 | Yes | * | 0.0211 | D-E |
